# Model of Production System Evaluation with the Influence of FDM Machine Reliability and Process-Dependent Product Quality

**DOI:** 10.3390/ma14195806

**Published:** 2021-10-04

**Authors:** Iwona Paprocka, Wojciech M. Kempa

**Affiliations:** 1 Department of Engineering Processes Automation and Integrated Manufacturing Systems, Faculty of Mechanical Engineering, Silesian University of Technology, Konarskiego 18A Str., 44-100 Gliwice, Poland; 2 Department of Mathematics Applications and Methods for Artificial Intelligence, Faculty of Applied Mathematics, Silesian University of Technology, 23 Kaszubska Str., 44-100 Gliwice, Poland; wojciech.kempa@polsl.pl

**Keywords:** predictive maintenance, fused deposition modeling, additive manufacturing, key process parameters, mean time to failure estimation, reliability of a machine, reliability characteristics, job shop system, ant-colony optimization

## Abstract

This paper investigates the Job Shop Scheduling Problem (JSSP) with FDM (Fused Deposition Modeling) machine unavailability constraints due to Predictive Maintenance (PdM) tasks, under the objective of minimizing the makespan, total tardiness and machine idle time. The Ant-Colony Optimization (ACO) algorithm is elaborated to deal with the JSSP. The reliability characteristics of the critical machine (FDM) influence the product as well as the production system quality. PdM periods are estimated based on historical data on failure-free times of the FDM machine components and deviations from the standards established for the key process parameters: infill density, layer thickness and extruder temperature. The standards for the key process parameters are identified based on investigation of the mechanical properties of printed elements. The impact of failure time and the number of nonstandard measurements of parameters on the quality of the Job Shop System (JSS) are observed. Failure rate of the FDM machine is corrected with the probability of a stoppage in the future period due to the “outlier” in measurements of any key parameters of the additive process. The quality robustness of production schedules increases with the disturbance-free operation of the FDM up to the peak value. After reaching the peak value the quality robustness decreases. The original issue of this paper is a model of scheduling production and maintenance tasks in a job shop system with an FDM machine as a bottleneck using ACO. Additionally, an original FDM-reliability model is also proposed. The model is based on weighted p-moving averages of the observed number of deviations from the norms, established for key process parameters such as fill density, layer thickness and extruder temperature.

## 1. Introduction

FDM (Fused Deposition Modeling) is the most widely used method of 3D printing. FDM can be used to make elements from thermoplastic polymers such as ABS (Acrylonitrile butadiene styrene), Nylon, PET (Polyethylene terephthalate) or HIPS (High Impact Polystyrene sheet) and elastomers such as TPU (Thermoplastic polyurethane) or TPE (Thermoplastic elastomer). Material in the form of a spool of filament is fed through an extruder to the printer head, where it is heated to a temperature where it becomes semiliquid. Then, it is extruded through a dye and laid out as a path with a width of 0.3 to 0.8 mm. The machine arranges paths next to each other, creating a single layer of material with a thickness of 0.05 to 0.4 mm. The model is built layer by layer and consequently becomes a solid. The disadvantages of this technology are the relatively long printing time, the need to create supports under the elements, or temperature shrinkage [1,2]. 

Elements printed in 3D technology are used not only as prototypes but can also be parts of other devices. An example may be the production of specialized scientific equipment. A great advantage is the possibility of obtaining semifinished products with unique properties, e.g., light, hollow semifinished products with spatial construction [3].

The FDM machine becomes critical when it is part of a job shop system. The limitations in the use of the FDM machine result from the long production time, the need to use printing material authorized by manufacturer, the diameter (typically 1.75 mm or 3 mm) and the extruding temperature (usually <250 °C) [3]. Additionally, a high cost of printing limits the application of this technology. The last disadvantage is the appearance of defects in the printed elements. 

There is a need to increase the repeatability of printed elements using the additive manufacturing method. Data generated by the FDM machine, data on the environment in which the FDM machine operates (temperature, humidity…), and printing process data at any stage of the additive manufacturing workflow must be analyzed to identify any relationship between the data and the quality of the printed item. The additive process can be controlled by having standards for the key additive process parameters.

The efficiency of a production system is influenced by both machine condition and quality of the manufactured product. Machine condition and component quality should be monitored and analyzed to prevent machine failure and poor product quality. Some studies have been conducted [4] to obtain an optimal buffer of inventory and inspection policy with preventive maintenance. The production system for a single type of product is subject to a random deterioration from an in-control state to an out-of-control state with a specific distribution. On-line inspection is started after some time to inspect products. Another human inspection is considered at the end of production cycle. Shortages are considered due to preventive maintenance. Meng et al. [5] investigated how the quality level, recovery (energy) cost and retail price impacted the product recovery decision: remanufacturing, directly reusing, and material recycling. Authors assumed that the quality distribution of each component was obtained by statistical model and life-cycle data analysis and was described by a uniform distribution. However, values of key quality parameters depend on the condition of a machine, environmental factors and human abilities and are typical for each type of machine and type of production process.

In the literature, the emphasis is put on forecasting a machine condition or on the influence of assumed process parameters, including quality, on efficiency criteria. This paper proposes a combination of preventive maintenance of FDM machines with effective and good-quality production including printed elements. The further study of the literature is divided into two sections: an overview of the reliability of FDM machines and an overview of production and maintenance scheduling.

### 1.1. Literature Review on the Reliability of FDM Machines

In the papers [6,7,8], parameters to control the additive process are defined: the FDM machine-generated data (e.g., technology, machine status, activity and sensor data), printing-part data (e.g., type of material, position and parameters), post-processing and quality-management data (e.g., the steps of post-processing and compliance requirements), data for reporting (e.g., inputs and outputs of the printing data and failure rate, throughput rate). 

According to Ćwikła [3], the key factor is the influence of parameters such as filling pattern, direction and density, coating thickness and printing temperature on selected mechanical properties of samples printed in 3D. According to Lyu and Manoochehri [9], three process parameters are important for FDM: extruder temperature, layer thickness and filling density. However, not only the dimensional accuracy of the manufactured part should be verified in order to assess the quality of the product. Additionally, the mechanical properties (strength parameters) of FDM 3D parts must be controlled in order to assess the quality of the product. Poor mechanical properties may result from poor mechanical strength of the material, but also from weak points between the layers, uncontrolled shrinkage during the cooling process, minor discontinuities in fiber extrusion, unevenness of the building platform and inaccuracies of extruder movements, etc. [3].

The additive process can be controlled in line with the acoustic emission standards for FDM machine elements. The authors [10,11] analyze the acoustic emission data of the FDM conditions in response to the need to develop an effective method of handling data. Acoustic emission wave signals are simplified first, then segmented, and the main components are analyzed to ensure product quality and reduce production costs. The hidden semi-Marcov model is used to model the state of the FDM. Lu and Chen [12] elaborated a model of degradation of machine-tooling components in order to overcome product quality deterioration. Online degradation data collected from sensors contain information about their unique degradation patterns. Tooling-component degradation rates are described by random variables whose posterior distributions are updated using degradation data based on the Bayesian approach. The real-time prediction of tooling component degradation is then used to predict the degradation of product quality and machine reliability based on a response model and integrated hazard function. Liu et al. [13] proposed an improved fault diagnosis approach based on acoustic emission sensors in the FDM process. The impacts of the acoustic emission obtained for the different states of the extruder are analyzed for the extraction of interesting features in the time and frequency domains in the signal segments. Feature dimension reduction based on linear discriminant analysis is used to reduce the feature space to only two dimensions, thus reducing the computational cost of classifying and identifying the condition.

The main advantage of this paper, and the main difference, is that predictive maintenance (PdM) periods are estimated based on historical data on failure-free times of the FDM machine components and deviations from the standards established for the key process parameters: infill density, layer thickness and extruder temperature. The standards for the key process parameters are identified based on investigation of the mechanical properties of printed elements. In this paper, an original FDM-reliability model is also proposed. The model is based on weighted p-moving averages of the observed number of deviations from the norms established for key process parameters such as fill density, layer thickness and extruder temperature.

### 1.2. Literature Review on Production and Maintenance Scheduling

The reliability characteristics of an FDM machine affect the quality of the product and the production system. Operating conditions should be analyzed for a single machine due to various key process parameters, different process covariates directly affecting the failure rate of the machine and the production system. Hu et al. [14] analyzed two-machines flow shop with constant- or variable-in-time machine failure rate and constant operating conditions. A genetic algorithm for joint production and preventive maintenance planning was proposed to minimize makespan. Liping et al. [15] developed hybrid genetic and taboo search algorithms to optimize the weighted function of two goals, makepan and schedule stability. The scheduling problem of dynamic job shop is considered with the random arrival of tasks and machine failures. Berrichi et al. [16] proposed an ant-colony optimization algorithm to solve the problem of joint production and maintenance scheduling. The best allocation of production tasks to machines and preventive maintenance periods of the production system depends on meeting production and maintenance goals. The remaining literature review, taking into account the types of scheduling problem, maintenance policies, optimization methods and objective functions, is presented in Table 1.

The main advantage of this paper, and the main difference, is the Job Shop Scheduling Problem (JSSP) with the FDM machine unavailability constraints due to Predictive Maintenance (PdM) tasks, under the objective of minimizing the makespan, total flow time, total tardiness and machine idle time. The Ant-Colony Optimization (ACO) algorithm is elaborated to deal with the JSSP. Failure rate of the FDM machine is corrected with the probability of a stoppage in the future period due to the “outlier” in measurements of any key parameters of the additive process. According to the authors’ knowledge, this is the first paper devoted to scheduling tasks in the job shop system and maintenance of the FDM machine.

This paper is organized as follows: problem definition, notation and assumptions are presented in Section 2. Reliability model of the job shop system is presented in Section 3. The ACO for Predictive–Proactive Scheduling Problem is presented in Section 4. The reliability of the FDM machine corrected with the parameters describing process-dependent product quality is predicted in Section 5. In Section 6, the impact of failure time and the number of ACO parameters on the quality of the Job Shop System (JSS) is observed.

## 2. Problem Definition, Assumptions and Notation

It is important to draw conclusions from historical data about the failure times of the FDM machine components. However, the reliability of the FDM machine components must be corrected using covariates that directly affect the failure rate. These covariates can describe machine state, printing-process conditions, or part printing conditions. This paper analyzes the influence of important parameters of the printing process on the quality of products, the reliability of the FDM machine and the JS system. Any variation in data on environment in which the FDM machine operates (temperature, humidity…), in data on printing process (sensor data on extruder temperature, machine motions, infill density, layer thickness), or in data of printing part (e.g., type of material, dimensional accuracy of the part) must be noticed. In order to determine product quality, interactions between environmental, machine or process data and the mechanical properties of the manufactured part must be identified.

The aim of this paper is to investigate the job shop system with FDM (Fused Deposition Modeling) machine unavailability constraints due to Predictive Maintenance (PdM) tasks, under the objective of minimizing the makespan, total tardiness and machine idle time. Additionally, the aim is to estimate the reliability characteristics of the critical machine (FDM) that influence the product as well as the production system quality. PdM periods are estimated based on historical data on failure-free times of the FDM machine components and deviations from the standards established for the key process parameters: infill density, layer thickness and extruder temperature.

The job shop (JS) system is dedicated to a number of tasks *t*, *τ* = 1,…, *t* with given assumptions:

- the workflow of each production task may be different, in other words, each process may route through different machines in a different order;
- non-reentrant case;
- the number of operations of each task equals the number of machines;
- each operation of a task is preassigned to a machine;
- each operation of a task is executed on a different machine;
- Production tasks are executed on a number of machines (resources) r, *r* = 1, *υ* with given constraints:
- pre-emptive case for the critical machine (machine can be interrupted);
- each machine can be an input and output of the workflow of a task;
- the FDM machine is the bottleneck;
- historical data on failure-free times of the FDM machine components and deviations from the standards established for the key process parameters: infill density, layer thickness and extruder temperature are given;
- machine setup times equal zero.

Moreover, the maintenance task is scheduled at the time of the expected failure of the FDM machine in the Gantt chart. The production tasks are scheduled in the order that the ant arrives. This is a scheduling problem with maintenance constraints. The problem of scheduling tasks in job shop systems is referred to as one of the complex optimization problems due to the time-consuming computational process. The complexity of the job shop scheduling problem increases with:

- the number of products;
- the number of machines;
- the frequency of machine failure and/or poor quality of the product.

The following notation was used to develop the model:

*t* the number of tasks, *τ* = 1,…, *t*
*υ* the number of machines (resources), *r* = 1,…, *υ*
*x_τ_* the number of operations of task *τ*, *o_τ_* = 1,…, *x_τ_*
*m* the number of parallel elements of FDM machine, i=1, …, m
n the number of scheduling periods, $\left[\left(j-1\right)T,\;jT\right)$, j=1, …, n,
*K_i,j_* the number of failures observed in the jth period, $k_{i,1},\;k_{i,2},\;\ldots ,\;k_{i,n}$
d the number of measurements of quantities E, L and F every $\frac{T}{d}$ time units,
E the extruder temperature
L the layer thickness
F the infill density
$\left[m_{E}-c\sigma_{E},m_{E}+c\sigma_{E}\right]$ norms for extruder temperature E, average value $m_{E}$ and standard deviation $c\sigma_{E}$ for extruder temperature E
$\left[m_{L}-c\sigma_{L},m_{L}+c\sigma_{L}\right]\;\mathrm{norms}\;\mathrm{for}\;$layer thickness L, average value $m_{L}$ and standard deviation $c\sigma_{L}$ for layer thickness L
$\left[m_{F}-c\sigma_{F},m_{F}+c\sigma_{F}\right]\;\mathrm{norms}\;\mathrm{for}\;$infill density *F*, average value $m_{F}$ and standard deviation $c\sigma_{F}$ for infill density *F*
$q_{i}^{\left(E\right)},\;q_{i}^{\left(L\right)}$ and $q_{i}^{\left(F\right)}$ are empirical probabilities for the first “outlier” (in measurement of E, L and F)
$X_{i,j,1},\;\ldots ,\;X_{i,j,K_{i,j}}$ failure-free times
$MTBF_{i}\;$Mean Time Between Failures for element i 
$MTTF_{i}\;$Mean Time To Failure for element i
$MTTR_{i}$ Mean Time of Repair for element i
MTBF Mean Time Between Failures for FDM machine
MTTF Mean Time To Failure for FDM machine
MTTR Mean Time of Repair for FDM machine
*RDT* real disturbance-free time FDM machine
RS Right Shifting heuristic
RPM Reschedule on Parallel Machines heuristic
MIDOS Minimal Impact of Disrupted Operation on the Schedule heuristic
$\mu_{i,j}$ parameter of exponentially distributed failure-free time of FDM machine
$\alpha_{i,n+1}$ parameter of exponentially distributed repair time for the element i
$e^{FDM}$ predefined mean repair time of the FDM machine in the planned period $\left[nT,\;\left(n+1\right)T\right).$
p the number of weights $w_{1},\;w_{2},\;\ldots ,\;w_{p}$ for averages of number of failures
*u* the predictive schedule (ant)
$u^{*}$ the reactive schedule
ϖ_1_, ϖ_2_, ϖ_3_, ϖ_4_ weights of predictive criteria,
z_1_, z_2_ weights of reactive criteria,
$C\left(u\right)$ the makespan criterion of predictive schedule (represented by an ant) *u*,
$Y\left(u\right)$ the total flow time of tasks executed in predictive schedule *u*,
$TD\left(u\right)$ the total delay of tasks,
$IT\left(u\right)$ the idle time of machines,
$FF\left(u\right)\;$the efficiency of the production system,
$SR\left(u*\right)$ the solution robustness of reactive schedule u∗,
$QR\left(u*\right)$ the quality robustness of reactive schedule u∗,
$SF\left(u*\right)\;$The reliability of the production system achieved by ant u*
$SF\left(u^{**}\right)$—the reliability of the schedule coded by the best ant u**
$tz_{x\tau }$ the due date of the last operation *x_τ_* of task *τ*,
$st_{1\tau }$ the start time of the first operation of task *τ*,
$st_{\tau ,o_{\tau }}\left(u\right)$ is start time of operation *o_τ_* of task *τ* in predictive schedule *u*;
$st_{\tau ,o_{\tau }}\left(u*\right)$ is the start time of operation *o_τ_* of task *τ* in reactive schedule u*.
$dl_{\tau }$ the deadline of task *τ*,
$mtz_{xrs\;}$—minimum due date of the last operation *x_rs_* of two adjacent tasks: *r* and *s*,
$DY_{\tau }$ the delay of task *τ*,
*np*—number of possible tasks to schedule after task *r*,

Moreover, variables and parameters of the ACO

A  the number of ants, *u* = 1,…, *A*
*B* the number of iterations,
β the relative importance between the pheromone trace and the reciprocal of the due date (distance)
*ρ* the pheromone evaporation factor ρ∈⟨0,1⟩,
*q* a random parameter from ⟨0,1⟩ which decides about exploration or exploration selection by an ant
V*T(u)* the vector of tasks randomly generated for coding the start position of ant *u*
*TL(u)* the taboo list of the ant *u*
$\eta =\frac{1}{tz_{xrs}}$ the reciprocal of the due date of task *s* scheduled after the task *r*
$N_{u}\left(r\right)\;$the set of those tasks that ant *u* (after scheduling task *r*) has not yet scheduled
$p_{u}\left(r,s\right)$ a variable randomly selected
*αe* the pheromone evaporation value, (1—*αe*) ϵ <0,1> is the glow of the pheromone
*mp* the number of ants that have passed the path from point *r* to point *s*
$\tau p\left(r,s\right)$ the actual amount of pheromone on the edge *eu* between points *r* and *s*/on the path from point *r* do *s*
$\Delta \tau p_{u^{**}}\left(r,s\right)$ the increase in the pheromone trace, $\left(r,s\right)\in L_{u^{**}}$
$L_{u^{**}}$ the sequence of production tasks belonging to the best ant u**

## 3. Reliability Modeling of the Job Shop System

### 3.1. The Model of the FDM Machine Reliability

The analyzed FDM machine consists of i parallel elements, i=1, …, m. The historical operation time of the machine is divided into n equal scheduling periods, $\left[\left(j-1\right)T,\;jT\right)$, j=1, …, n, where T>0 is fixed. For the ith element *K_i,j_* denotes the number of failures observed in the jth period $\left[\left(j-1\right)T,\;jT\right)$, and $X_{i,j,1},\;\ldots ,\;X_{i,j,K_{i,j}}$ denote successive failure-free times in this period. For each historical period the distribution parameters are estimated in order to describe the phenomenon of failure rate of the FDM machine element. 

Let us assume that for fixed i=1, …, m and j=1, …, n the failure-free times $X_{i,j,1},\;\ldots ,\;X_{i,j,K_{i,j}}$ are independent random variables exponentially distributed with mean $\mu_{i,j}^{-1}$, so with the PDF of the form

(1) $$f_{i,j}\left(t\right)=\left\{\begin{array}{cc} \mu_{i,j}exp\left(-\mu_{i,j}t\right), & t>0\\ 0, & t\leq 0. \end{array}\right.\;$$

Obviously, in general, parameters $\mu_{i,j}$ can be different for different i and j. To estimate them we use the maximum likelihood method that gives the following form of the estimator of $\mu_{i,j}$:

(2) $${\tilde{\mu }}_{i,j}=\frac{k_{i,j}}{\sum_{r=1}^{k_{i,j}}x_{i,j,r}},$$

where $k_{i,j}$ and $x_{i,j,r}$ are empirically observable values of random variables $K_{i,j}$ and $X_{i,j,r}.$

Having estimators ${\tilde{\mu }}_{i,j}$ for fixed i=1, …, m and for successive j=1, …, n, we predict the value $\mu_{i,n+1},$ namely the value of the parameter of exponentially distributed single failure-free time in the planned period $\left[nT,\;\left(n+1\right)T\right)$. We propose here two different methods of estimation:

- a classical last-square linear regression;
- a linear regression based on weighted moving averages.

In the latter approach, having physically observed number of failures $k_{i,1},\;k_{i,2},\;\ldots ,\;k_{i,n}$ of the element i in successive periods $\left[\left(j-1\right)T,\;jT\right),$ where j=1, …, n, we define weighted *p*-moving averages in the following way

$${\bar{k}}_{i}^{\left(1\right)}=\frac{1}{p}\sum_{j=1}^{p}k_{i,j}w_{j},\;\;{\bar{k}}_{i}^{\left(2\right)}=\frac{1}{p}\overset{p+1}{\underset{j=2}{\sum }}k_{i,j}w_{j-1},\;{\bar{k}}_{i}^{\left(n-p+1\right)}=\frac{1}{p}\overset{n}{\underset{j=n-p+1}{\sum }}k_{i,j}w_{j-n+p},$$

where $p<\frac{n}{2}$ is an odd number and weights $w_{1},\;w_{2},\;\ldots ,\;w_{p}$ are chosen in such a way that

$$w_{1}\leq w_{2}\leq \ldots \;\leq \;w_{p}$$

and, naturally, $\overset{p}{\underset{j=1}{\sum }}w_{p}=1.$ Thus, the “youngest” observations are of the greatest importance. Next, having values ${\bar{k}}_{i}^{\left(1\right)},\;\ldots ,\;{\bar{k}}_{i}^{\left(n-p+1\right)},\;$we predict ${\bar{k}}_{i}^{\left(n-p+1\right)}$ using classical least-square regression. 

Now, due to the fact that

$${\bar{k}}_{i}^{\left(n-p+2\right)}=\frac{1}{p}\overset{n+1}{\underset{j=n-p+2}{\sum }}k_{i,j}w_{j-n+p-1}=\frac{1}{p}\overset{n}{\underset{j=n-p+2}{\sum }}k_{i,j}w_{j-n+p-1}+\frac{1}{p}k_{i,n+1}w_{p},$$

Denoting

(3) $$a_{i,p}\left(n\right)=\frac{1}{p}\sum_{j=n-p+2}^{n}k_{i,j}w_{j-n+p-1},\;$$

(this value can be computed via observable numbers of failures in historical periods), we obtain the predicted number of failures in the planning period, given as

(4) $$k_{i,n+1}=p\left({\bar{k}}_{i}^{\left(n-p+2\right)}-a_{i,p}\left(n\right)\right).$$

Since failure-free times are assumed to have exponential distributions, we also predict the value of parameter $\mu_{i,n+1}$ as

(5) $${\tilde{\mu }}_{i,n+1}=\frac{k_{i,n+1}}{T}$$

Assuming that, all elements of the FDM machine are in series, the reliability of the machine is computed by the product of reliabilities of each its element i, so

(6) $$R^{FDM}\left(t\right)=\overset{m}{\underset{i=1}{\prod }}R_{i}\left(t\right)=\overset{m}{\underset{i=1}{\prod }}\overset{\infty }{\underset{t}{\int }}f_{i,j}\left(u\right)du=\overset{m}{\underset{i=1}{\prod }}e^{-\mu_{i,n+1}t}=\exp\left(-t\overset{m}{\underset{i=1}{\sum }}\mu_{i,n+1}\right)$$

After obtaining the predicted values of the distribution parameter ${\tilde{\mu }}_{i,n+1}$ for each element *i* for the planning period, reliability characteristics are computed as follows:

- Mean Time Between Failures (MTBF) for element i (equal to Mean Time To Failure for element i + Mean Time of Repair (MTTR) for element i)

(7) $$MTBF_{i}=MTTF_{i}+MTTR_{i}=E\left(X_{i,n+1,1}+Y_{i,n+1,1}\right)=\frac{1}{{\tilde{\mu }}_{i,n+1}}+\frac{1}{\alpha_{i,n+1}}$$

where $\alpha_{i,n+1}$ is the predefined value of the parameter of exponentially distributed repair time for the element i in the planned period $\left[nT,\;\left(n+1\right)T\right).$

- Mean Time Between Failures for the FDM machine (equal to Mean Time To Failure for the FDM machine + Mean Time of Repair for the FDM machine)

(8) $$MTBF=MTTF+MTTR=E\left(U_{n+1,1}+W_{n+1,1}\right)=\int_{0}^{t}R^{FDM}\left(y\right)dy+e^{FDM}$$

where $e^{FDM}$ is the predefined mean repair time of the FDM machine in the planned period $\left[nT,\;\left(n+1\right)T\right).$

### 3.2. The Model of the Process-Dependent Product Quality 

The fabricated product quality can vary with time. In order to estimate the quality of the product, its mechanical properties should be analyzed. Process parameters influence the quality of the fabricated product, especially the extruder temperature E, the layer thickness L and the infill density F [9]. 

The model of predicting quality of the fabricated product is based on the comparative analysis of mechanical properties of printed elements and process parameters. 

Let us assume that quantities E, L and F are random variables with truncated normal distributions with parameters (mean value, standard deviation) $\left(m_{E},\sigma_{E}\right),\;\left(m_{L},\sigma_{L}\right)\;$and $\left(m_{F},\sigma_{F}\right),$ respectively. So, we have

(9) $$E\left(t\right)≝P\left\{E<t|E>0\right\}=\frac{P\left\{0<E<t\right\}}{P\left\{E>0\right\}}=\frac{\Phi \left(\frac{t-m_{E}}{\sigma_{E}}\right)-\Phi \left(-\frac{m_{E}}{\sigma_{E}}\right)}{1-\Phi \left(-\frac{m_{E}}{\sigma_{E}}\right)}$$

(10) $$L\left(t\right)≝P\left\{L<t|L>0\right\}=\frac{P\left\{0<L<t\right\}}{P\left\{L>0\right\}}=\frac{\Phi \left(\frac{t-m_{L}}{\sigma_{L}}\right)-\Phi \left(-\frac{m_{L}}{\sigma_{L}}\right)}{1-\Phi \left(-\frac{m_{L}}{\sigma_{L}}\right)}$$

(11) $$F\left(t\right)≝P\left\{F<t|F>0\right\}=\frac{P\left\{0<F<t\right\}}{P\left\{F>0\right\}}=\frac{\Phi \left(\frac{t-m_{F}}{\sigma_{F}}\right)-\Phi \left(-\frac{m_{F}}{\sigma_{F}}\right)}{1-\Phi \left(-\frac{m_{F}}{\sigma_{F}}\right)}$$

where $\Phi \left(\cdot \right)$ stands for the cumulative distribution function of the standard normal distribution and t>0.

Let us assume that particular parameters meet the standards if their measured values do not deviate from the mean value by more than c times the standard deviation, where $c\in \left[1,3\right].$ The norms for individual parameters are therefore determined by the following inequalities:

- extruder temperature E: $\left[m_{E}-c\sigma_{E},m_{E}+c\sigma_{E}\right],$
- layer thickness L: $\left[m_{L}-c\sigma_{L},m_{L}+c\sigma_{L}\right],$
- infill density F: $\left[m_{F}-c\sigma_{F},m_{F}+c\sigma_{F}\right].$

As previously, we investigate the quality of the fabricated product on n historical periods, namely in $\left[\left(j-1\right)T,\;jT\right),$ where j=1, …, n. In each of these periods, we make d measurements of quantities E, L and F every $\frac{T}{d}$ time units, observing whether each of the three parameters is within the established norm (range). For example, for the period $\left[0,T\right)$ we measure at times

$$0,\frac{T}{d},\;\frac{2T}{d},\;\ldots ,\;\frac{\left(d-1\right)T}{d},$$

where d is a predefined positive integer.

In fact, we are interested in the first moment of measurement in a given period $\left[\left(j-1\right)T,\;jT\right),$ when the result is outside the norm. We introduce the following notations:

$$q_{i}^{\left(E\right)}=\frac{number\;of\;periods\;in\;which\;E\;is\;out\;of\;the\;norm\;at\;the\;ith\;measurement\;for\;the\;first\;time}{n},$$

$$q_{i}^{\left(L\right)}=\frac{number\;of\;periods\;in\;which\;L\;is\;out\;of\;the\;norm\;at\;the\;ith\;measurement\;for\;the\;first\;time}{n},$$

and

$$q_{i}^{\left(F\right)}=\frac{number\;of\;periods\;in\;which\;F\;is\;out\;of\;the\;norm\;at\;the\;ith\;measurement\;for\;the\;first\;time}{n},$$

where i=0, …, d−1 (we identify the measurement at $\left(j-1\right)T$ as the 0th one).

So $q_{i}^{\left(E\right)},\;q_{i}^{\left(L\right)}$ and $q_{i}^{\left(F\right)}$ are empirical probabilities that the first “outlier” (in measurement of E, L and F) occurs at the ith measurement in a single period$\;\left[\left(j-1\right)T,\;jT\right)$, respectively.

Consequently, we can estimate the probability of stopping of a single machine in the planned period $\left[nT,\;\left(n+1\right)T\right)$ due to the “outlier” in measurement of E, L and F, respectively, after time $t\in \left[0,\;T\right)$ as

$$\overset{d-1}{\underset{i=t+1}{\sum }}q_{i}^{\left(E\right)},\;\overset{d-1}{\underset{i=t+1}{\sum }}q_{i}^{\left(L\right)},\;\overset{d-1}{\underset{i=t+1}{\sum }}q_{i}^{\left(F\right)},\;$$

respectively.

### 3.3. The Model of the Production System Reliability and Efficiency

Because the FDM machine must be stopped when the failure occurs or at least one of parameters E, L and F will be out of the norm, then the probability of stopping a single element of a machine after time $t\in \left[0,\;T\right)$ in the planned period can be expressed as

(12) $$S_{j}\left(t\right)≝P\left\{stopping\;an\;element\;j\;of\;a\;machine\;after\;time\;t\right\}=e^{-\mu_{j,n+1}}\cdot \overset{d-1}{\underset{i=t+1}{\sum }}q_{i}^{\left(E\right)}\cdot \overset{d-1}{\underset{i=t+1}{\sum }}q_{i}^{\left(L\right)}\cdot \;\overset{d-1}{\underset{i=t+1}{\sum }}q_{i}^{\left(F\right)},$$

where j=1, …, m **and** 0≤t<T.

Consequently, the probability of stopping the whole machine, consisting of m independent elements, can be computed as

(13) $$P\left\{stopping\;a\;machine\;after\;time\;t\right\}=\overset{m}{\underset{j=1}{\prod }}S_{j}\left(t\right)$$

where 0≤t<T. The maintenance task is planned for the time of the expected disturbance of the FDM machine operation due to the increased probability of failure and the probability of exceeding the permissible (beyond the standard) quality parameters. The efficiency of the production system is evaluated using the objective function (14)

(14) $$FF\left(u\right)=\varpi_{1}\cdot C\left(u\right)+\varpi_{2}\cdot Y\left(u\right)+\varpi_{3}\cdot TD\left(u\right)+\varpi_{4}\cdot IT\left(u\right),$$

(15) $$C\left(u\right)=\max\left[tz_{x\tau }\right],$$

(16) $$Y\left(u\right)=\overset{t}{\underset{\tau =1}{\sum }}\left(tz_{x\tau }-st_{1\tau }\;\right),$$

(17) $$TD\left(u\right)=\overset{t}{\underset{\tau =1}{\sum }}\left[0,DY_{\tau }\right],\;where\left\{\begin{array}{c} 0,\;\;if\;dl_{\tau }-tz_{x\tau }\leq 0\\ DY_{\tau },\;\;if\;dl_{\tau }-tz_{x\tau }>0, \end{array}\right.$$

(18) $$IT\left(u\right)=\overset{\upsilon }{\underset{r=1}{\sum }}I_{r}.$$

A reactive schedule is generated to check how the FDM failure affects the predictive schedule. Thanks to the proactive approach, which is based on generation and testing, the best predictive and proactive schedule is adopted for implementation. The newly generated reactive schedule should replicate the previous one as much as possible, making minimal changes due to the FDM failure. The reliability of the production system is assessed using the weighted function of solution robustness (SR) and quality robustness (QR)

(19) $$SF\left(u^{*}\right)=z_{1}\cdot SR\left(u^{*}\right)+z_{2}\cdot QR\left(u^{*}\right),$$

(20) $$SR\left(u*\right)=\overset{t}{\underset{\tau =1}{\sum }}\overset{x_{\tau }}{\underset{o_{\tau }=1}{\sum }}\left|st_{\tau ,o_{\tau }}\left(u\right)-st_{\tau ,o_{\tau }}\left(u*\right)\right|,$$

(21) $$QR\left(u^{*}\right)=\left|FF\left(u\right)-FF\left(u^{*}\right)\right|,$$

(22) $$FF\left(u*\right)=\varpi_{1}\cdot C\left(u*\right)+\varpi_{2}\cdot Y\left(u*\right)+\varpi_{3}\cdot TD\left(u*\right)+\varpi_{4}\cdot IT\left(u*\right).$$

Reactive Gantt charts (schedules) u * are built using two heuristics:

(1) Right shift (RSh) of interrupted operations when there is only one FDM machine and machine components can be calibrated to continue the printing process. Additionally, subsequent tasks are shifted to the right when the priority constraint is violated;
(2) Rescheduling the interrupted task and successive tasks on the first available parallel machines (RDO) in the case of poor product quality or failure of the FDM machine, provided that available FDM parallel machines also exist.

The process of building failure-free schedules with good-quality products begins with generating basic schedules using the Ant-Colony Optimization Algorithm ACO [21]. Knowledge gained about reliability of key process parameters and the FDM machine is used to generate predictive schedules. The task for the key process parameters calibration or maintenance of the FDM machine is scheduled at the time indicated by the reliability characteristic: Mean Time To Failure. The ACO algorithm for predictive–proactive planning was coded in Borland C ++ and is presented in the next section. The graphical representation of the problem is also presented.

## 4. ACO for Predictive–Proactive Scheduling Production 

The method of predictive–reactive scheduling presented in [24] uses the advantage of computer simulation by repeating steps:

(3) generating a set of the best ant population using the makespan criterion for local and global update of the pheromone trace,
(4) building predictive schedules using the Minimal Impact of Disrupted Operation on the Schedule (MIDOS) rule for the best ant population (coded by ants). Only the data on failure-free times is analyzed in order to gain the knowledge on the Mean Time To Failure.
(5) assessment of the impact of a machine failure on reactive schedules using the weighted function of two: solution robustness (SR) and quality robustness (QR).
(6) selection of the best schedule (ant) using the weighted function of solution robustness and quality robustness.

The method of predictive–proactive scheduling presented in this paper uses the advantage of computer simulation by repeating steps:

(1) generating a set of the best ant population using the weighted function FF(u) of four criteria: makespan, total flow time, total tardiness and idle time of machines for global update of the pheromone trace,
(2) building predictive schedules using historical information on failure numbers of the FDM machine and numbers of abnormal values of key quality-process parameters. On-time information on the key process parameters are read from sensors. The maintenance task is scheduled for the duration of the anticipated disturbance of the FDM machine’s operation for each ant for global update,
(3) assessment of the impact of a machine disturbance on reactive schedules using the weighted function of both: solution robustness (SR) and quality robustness (QR) for global update,
(4) selection of the best schedule (ant) using the weighted function of solution robustness and quality robustness.

Steps of the ACO algorithm for generating predictive–proactive schedules in job shop scheduling problems are presented in the following subsections.

### 4.1. Ants Coding

A random task τ is selected from a vector of tasks VT(u) randomly generated for coding the start position of ant u. The position (task) is memorized in the taboo list of the ant TL(u). Next, the ant can select two neighboring tasks of τ from list VT(u).

### 4.2. Solution Generation

If q ≤ 0.5, the exploitation step is selected, otherwise the exploration step is generated. Each selected task is saved in the Taboo list TL(u) and scheduled in the Gantt chart. The probability of ant u moving from task r to task s is computed using (24) for exploration or (23) for exploitation step. With each move, the ant reduces the value of the pheromone information on the track (r, s) (10) [3]. In the job shop scheduling problem, L_nn_ is stated as the minimum time for completing a task after scheduling all tasks from the neighborhood. Value n is stated as the number of tasks in the neighborhood of ant u. The final solution coded by ant *u* is presented by the production-task sequence achieved in Taboo list TL(u).

(23) $$AS=\left\{\begin{array}{c} arg\underset{eu\in N_{u}\left(r\right)}{max}\left\{\right.\left.\left[\tau p\left(r,eu\right)\right]\cdot {\left[\eta \left(r,eu\right)\right]}^{\beta }\;\right\},\;for\;q\leq 0.5\;\\ p_{u}\left(r,s\right),\;for\;q>0.5 \end{array}\right.$$

(24) $$p_{u}\left(r,s\right)=\left\{\begin{array}{c} \frac{\left[\tau p\left(r,s\right)\right]\cdot {\left[\eta \left(r,s\right)\right]}^{\beta }}{\sum_{eu\in N_{u}\left(r\right)}\left[\tau p\left(r,s\right)\right]\cdot {\left[\eta \left(r,s\right)\right]}^{\beta }},\;for\;s\in N_{u}\left(r\right)\\ 0,\;for\;s\notin N_{u}\left(r\right) \end{array}\right.$$

### 4.3. Local Update of the Pheromone Trace for Due-Date Optimization

Local updating of the pheromone trace means that ants do not focus on one path (sequence of production tasks) and choose other solutions more willingly. The pheromone trace is reduced by the amount of $\Delta \tau p_{u}\left(r,s\right)$ in the equation

(25) $$\tau p\left(r,s\right)\leftarrow \left(1-\rho \right)\cdot \tau p\left(r,s\right)+\rho \cdot \Delta \tau p_{u}\left(r,s\right),$$

(26) $$\Delta \tau p_{u}\left(r,s\right)=\frac{1}{np\cdot mtz_{xrs\;}}$$

### 4.4. The Predictive–Reactive Schedule Generation

The decoding procedure begins after each ant receives a production-task sequence. In the procedure of predictive schedule generation, production jobs are allocated to machines in the order resulting from the production job sequence, taking into account the FDM-machine occupancy limitation for the maintenance task. After the disturbance, a rescheduling procedure is used for generating a reactive schedule: Right Shifting (RS) or Reschedule on Parallel Machines (RPM). The predictive schedule encoded by each ant is tested for the need to stop the FDM machine due to failure or poor quality of process parameters.

### 4.5. Global Update of the Pheromone Trace for Stability and Robustness Optimization

The global update of pheromones takes place at the edges of the best path from the anthill to food. The best path encodes the best schedule into the predictive–proactive problem obtained for each iteration. The pheromone trace is increased by the amount of $\Delta \tau p_{u^{**}}\left(r,s\right)$ in Equation

(27) $$\tau p\left(r,s\right)\leftarrow \left(1-\alpha e\right)\cdot \tau p\left(r,s\right)+\alpha \cdot \overset{mp}{\underset{u=1}{\sum }}\Delta \tau p_{u^{**}}\left(r,s\right),$$

(28) $$\Delta \tau p_{u^{**}}\left(r,s\right)=\left\{\begin{array}{c} \frac{1}{SF\left(u^{**}\right)},\;for\;\left(r,s\right)\in L_{u^{**}}\\ 0,\;for\;\left(r,s\right)\notin L_{u^{**}} \end{array}\right.$$

### 4.6. The Best Predictive–Proactive Schedule Selection 

Solution robustness SR (20) informs the decision maker of to what extent the reactive schedule differs from the predictive schedule. Four criteria are used to assess the robustness of the quality of the reactive schedule QR (21): makespan, total flow time, total delay, and machine idle time. The best solution with the minimum value of the weighted function SR and QR (19) is adopted for implementation in the job shop production system. The pheromone information is updated for each path taken by the best ant (27). 

## 5. Reliability Characteristics Prediction

The historical operation time of the FDM machine is divided into n=35 equal scheduling periods, $\left[\left(j-1\right)T,\;jT\right)$, j=1, …, 35, where T=1000 h. Data on number of failures, *K_j,_* observed in the jth period, and successive failure-free times $X_{j,1},\;\ldots ,\;X_{j,K_{j}}$ of the FDM machine for period *j* is presented in [25]. Repair time of the FDM machine is described by exponential distributions with α_36_ = 0.5, thus the expected distribution value (the mean repair time) equals 2 h. 

In the first approach, we assume that for j=1, …, 35 the failure-free times $X_{j,1},\;\ldots ,\;X_{j,K_{j}}$ are independent random variables, exponentially distributed with mean $\mu_{j}^{-1}$ (1). For each historical period the distribution parameters are estimated in order to describe the phenomenon of failure rate of the FDM machine element using the maximum likelihood method (2). The prediction of the parameter for period 36 is described by the equation μ˜36=0.000003∗36^2−0.00006∗36+0.0091. Mean Time To Failure equals 92.35 h for the FDM machine.

In the latter approach, only the number of failures $k_{1}=9,\;k_{2}=7,\;\ldots ,\;k_{n}=10$ of the FDM machine in successive periods $\left[\left(j-1\right)T,\;jT\right),$ where j=1, …, 35 is analyzed. Weighted *p*-moving averages in the following way

$${\bar{k}}_{i}^{\left(1\right)}=8.5,\;{\bar{k}}_{i}^{\left(2\right)}=8.51\text{…}{\bar{k}}_{i}^{\left(32\right)}=7.5282$$

where weights are chosen in such a way that

$$w_{1}=0.1\leq w_{2}=0.2\leq w_{3}=0.2\leq w_{4}=0.25\leq w_{5}=0.25.$$

Next, having values ${\bar{k}}_{i}^{\left(1\right)},\;\ldots ,\;{\bar{k}}_{i}^{\left(n-p+1\right)},\;$we predict ${\bar{k}}_{i}^{\left(36\right)}=16.8046$ using classical least-square regression. Since failure-free times are assumed to have exponential distributions, we also predict the value of parameter $\mu_{i,n+1}$ as ${\tilde{\mu }}_{i,n+1}=0.0148.\;$Mean Time To Failure for the FDM machine ${\mathrm{MTTF}}_{i}=59.5075.\;$

Let us consider the influence of the infill density parameter, that is percentage of infill of the space inside perimeters and solid layers [3]. We assume that filling density standard F: [0.4–0.1, 0.4 + 0.1] was determined by conducting empirical tests [3]. The fill parameter is responsible for the fill density. With higher values it also affects the fill pattern without affecting the outer layers of the print. All specimen groups used to test the influence of the infill density parameter were printed for steady characteristics, such as perimeters, solid layers, fill pattern and extrusion multiplier. Experiments were conducted for various infill densities, namely 10%, 25%, 40% and 60%. The slicer’s default value of the infill density is usually 0.3–0.4. As a result of the conducted experiments, the following conclusion was given: the increase in infill value reduces deformation. In other words, the strength of the sample grows with the infill density. The conducted experiments also summarized that it is cost effective to reduce the fill density to about 40–50%. For small and complex parts, it is reasonable to increase this parameter to about 0.6. Further increasing the value of the infill density parameter raises the pressure in the extruder, which forces the reduction of printing speed.

Let us analyze the quality of the fabricated product in each historical period j=1, …, 35. In each of these periods, d=1000 measurements of the infill density F are collected. Every 60 min, it is noted whether the filling density parameter is within the established standard [0.4–0.1; 0.4 + 0.1]. Numerical analysis is conducted using MS Excel. 

Empirical probabilities $q_{i}^{\left(F\right)}$ that the first “outlier” (in measurement of F) occurs at the ith measurement in a single period$\;\left[\left(j-1\right)T,\;jT\right)$ are

$$q_{1}^{\left(F\right)}=0.02857,\;q_{2}^{\left(F\right)}=0,q_{3}^{\left(F\right)}=0.02857,q_{4}^{\left(F\right)}=0.085714,\;q_{5}^{\left(F\right)}=0.02857,q_{6}^{\left(F\right)}=0.05714,q_{7}^{\left(F\right)}=0,q_{8}^{\left(F\right)}=\;0.05714,q_{9}^{\left(F\right)}=0.02857,q_{10}^{\left(F\right)}=0,q_{11}^{\left(F\right)}=0.02857,q_{12}^{\left(F\right)}=0.2,q_{13}^{\left(F\right)}=0.05714,q_{14}^{\left(F\right)}=0,q_{15}^{\left(F\right)}=0.05714,q_{16}^{\left(F\right)}=\;0.02857,q_{17}^{\left(F\right)}=0,q_{18}^{\left(F\right)}=0,\;q_{19}^{\left(F\right)}=0.05714,\;q_{20}^{\left(F\right)}=0.02857\ldots$$

In consequence, we can estimate the probability of stopping of the FDM machine in the planned period $\left[nT,\;\left(n+1\right)T\right)$ due to the “outlier” in measurement of F, after time $t\in \left[0,\;T\right)$ as

$$\overset{1000-1}{\underset{i=59+1}{\sum }}q_{i}^{\left(F\right)}=71\%,\;$$

## 6. Computer Simulation Results and Discussion

The impact of key process parameters on the operation time of a single production system is analyzed, for example the influence of uptime of the FDM machine. Different inputs and operating conditions characterize different production systems, therefore data analysis and computer simulations are performed for a single case study. Additionally, the impact of key ACO parameters on the operation time (makespan), total flow time of production tasks, total delay of tasks, idle time of machines, schedule robustness and quality robustness of the production system is analyzed.

### 6.1. The Impact of Key Process Parameters 

**Figure 1:**
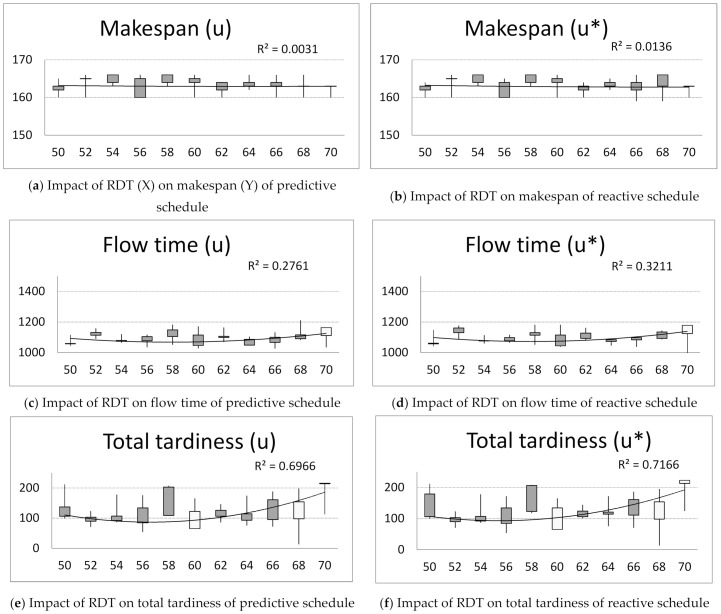

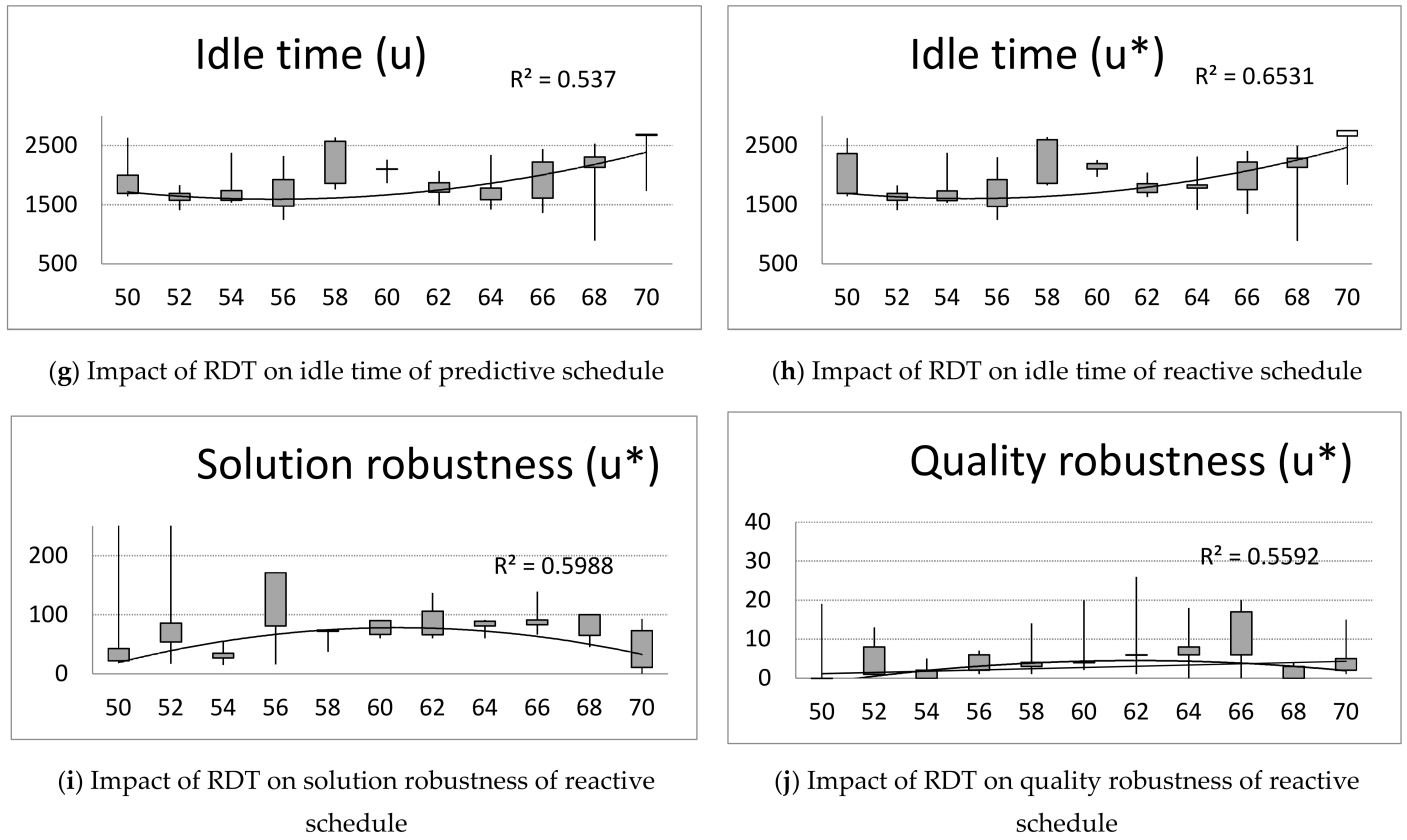
The impact of the FDM machine disturbance-free time RDT on predictive–reactive schedules assessed using the makespan C, total tardiness TD, flow time Y, idle time IT, solution robustness SR and quality robustness QR.

Computer simulations are run in order to investigate the influence of disturbance-free time of the FDM machine over reliability of the job shop scheduling system described by 11 processes and 10 machines (11 × 10). Computer simulations are run for parameters of the ACO: parameter of the relative importance between the pheromone trace and the reciprocal of the distance β=1; pheromone evaporation factor $\rho =\left\{0.6\right\};$ number of ants, A = {15}; number of iterations, B = {20} and parameter q which decides on exploration or exploration selection by an ant, $q=\left\{0.5\right\}.$ The ACO is run 5 times for changing real disturbance-free times, RDT, of the FDM machine {50, 52, …, 70} and for the Mean Time To Failure, MTTF = 59 and Mean Time of Repair, MTTR = 2. The maximum value, minimum value, first and third quintile of the obtained schedules for makespan *C*, total tardiness TD, flow time Y, idle time IT, solution robustness SR and quality robustness QR are presented in Figure 1. The x-axis represents the actual disturbance-free times of the FDM machine, X*_m_*_+1,1_ = {50, 52,…, 70}. The y-axis represents values of criteria of predictive schedules (u) and reactive schedules (u*). 

Analyzing the impact of failure time of the FDM machine and the number of cases of noncompliance with the key process parameters of the standards on makespan, flow time, total tardiness and idle time of predictive and reactive schedules, the following phenomenon can be noticed:

The disturbance-free operation of the FDM machine has little impact on the makespan criterion (Figure 1a,b).

The flow time of schedules increases with increasing the disturbance-free operation of the FDM machine (Figure 1c,d).

The total tardiness decreases with increasing the disturbance-free operation of the FDM. After reaching the peak value of RDT = 58, the total delay increases with the failure-free time of the FDM machine (Figure 1e,f).

The idle time increases with the disturbance-free operation of the FDM. (Figure 1g,h).

Next, the impact of the FDM machine uptime on: the solution robustness and quality robustness is investigated (Figure 1g,h). The following phenomenon can be noticed: The solution robustness of predictive schedules decreases with the disturbance-free operation of the FDM machine. After reaching the peak value of RDT = 60, the solution robustness increases with the failure-free time of the FDM machine (Figure 1g).

The quality robustness increases with the disturbance-free operation of the FDM (Figure 1h).

### 6.2. The Impact of Key ACO Parameters 

Next, computer simulations were run in order to investigate the influence of key ACO parameters on the ability of achieving robust schedules for the job shop scheduling system (11 × 10). Computer simulations of the ACO were run for the pheromone evaporation factor ρ = {0.2, 0.3, 0.4, 0.5, 0.6, 0.7} number of ants, A = {5, 10, 15, 20, 25, 30}; number of iterations, B = {5, 10, 15, 20, 25, 30} and parameter q = {0.3, 0.4, 0.5, 0.6, 0.7, 0.8}. The ACO is run 10 times for each set of key ACO parameters and predicted failure time of the FDM machine, RDT = 70 and MTTF = 59 and MTTR = 2.

**Figure 2:**
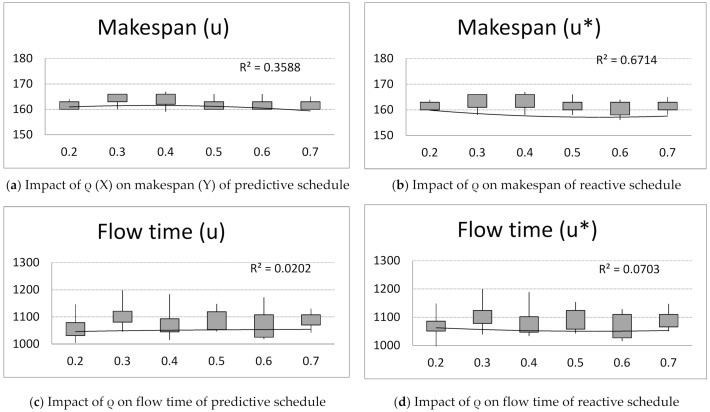

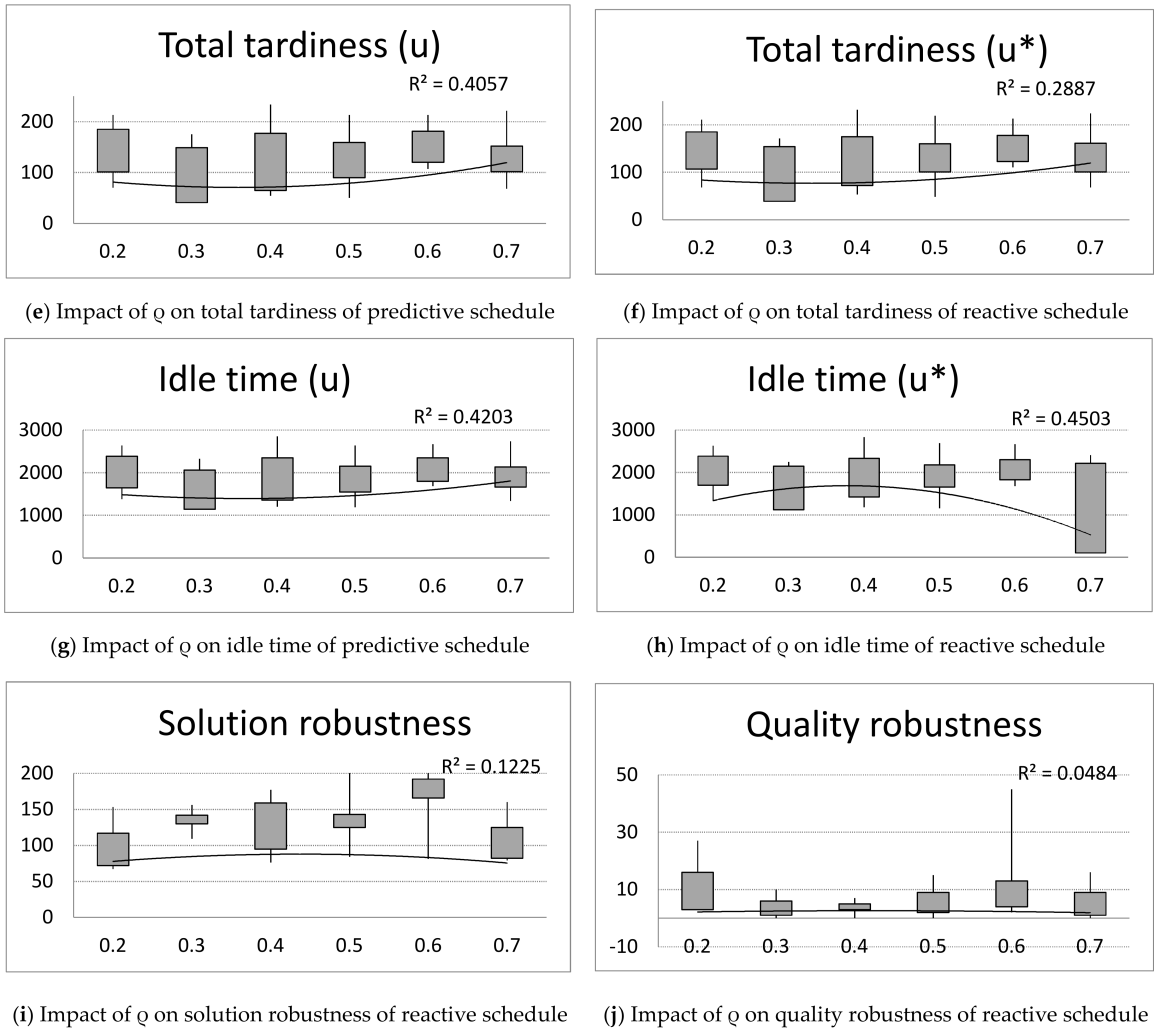
The impact of pheromone evaporation factor ρ = {0.2, 0.3, 0.4, 0.5, 0.6, 0.7} on makespan, flow time, total tardiness, and idle time of predictive schedules (u) and reactive schedules (u*) and solution robustness and quality robustness.

First, computer simulations were run for an unchanging number of ants A = 15, number of iterations B = 20, parameter q = 0.5 and changing pheromone evaporation factor α = ρ = {0.2, 0.3, 0.4, 0.5, 0.6, 0.7}. Observing the achieved values of criteria: makespan, flow time, total tardiness and idle time for predictive and reactive schedules (Figure 2), the following conclusion can be drawn:

The pheromone evaporation factor ρ has little impact on the makespan criterion (Figure 2a,b). The makespan increases with ρ. After reaching the peak value of ρ = 0.4, the makespan decreases with ρ for predictive schedule (Figure 2a). The inverse phenomenon can be noticed for reactive schedules (Figure 2b).

The flow time of schedules slightly increases with ρ for predictive schedules (Figure 2b). The flow time of schedules slightly decreases with ρ for reactive schedules (Figure 2c).

The total tardiness decreases with ρ. After reaching the peak value of ρ = 0.3, the total delay increases with ρ (Figure 2e,f).

The idle time decreases with increasing value of ρ. After reaching the peak value of ρ = 0.4, the idle time increases with ρ for predictive schedule (Figure 2g). The inverse phenomenon can be noticed for reactive schedules (Figure 2h).

Next, the impact of the pheromone evaporation factor ρ on the solution robustness and quality robustness is investigated (Figure 2g,h). The following phenomenon can be noticed: The solution robustness of predictive schedules decreases with increasing value of ρ. After reaching the peak value for ρ = 0.4, the solution robustness increases with the value of ρ (Figure 2g).

The pheromone evaporation factor, ρ, has little impact on the quality robustness (Figure 2h).

**Figure 3:**
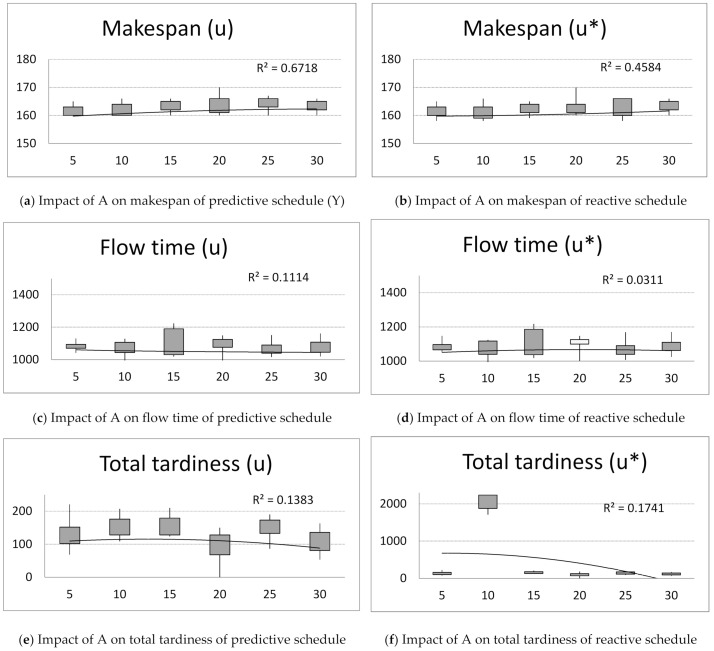

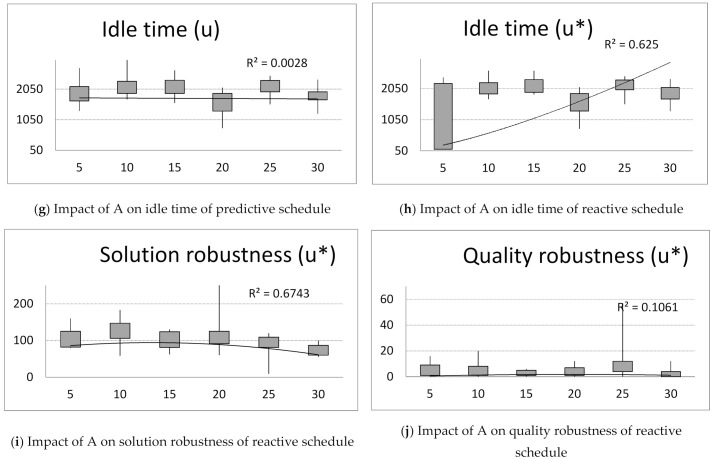
The impact of number of ants, A = {5, 10, 15, 20, 25, 30}; on makespan, flow time, total tardiness, and idle time of predictive schedules (u) and reactive schedules (u*) and solution robustness and quality robustness.

Next, computer simulations were run for unchanging number of iterations B = 20, parameter q = 0.5, pheromone evaporation factor ρ = 0.7 and changing number of ants, A = {5, 10, 15, 20, 25, 30}. Observing the achieved values of criteria: makespan, flow time, total tardiness and idle time for predictive and reactive schedules (Figure 3), the following conclusion can be drawn: 

The size of ant population A has little impact on the makespan criterion (Figure 3a,b). The makespan increases with A. 

The size of ant population A has little impact on the flow time criterion (Figure 3c,d). 

The flow time of schedules slightly decreases with ρ for predictive schedules (Figure 3c). 

The total tardiness decreases with increasing size of ant population A (Figure 3e,f).

The size of ant population A has little impact on the idle time criterion of predictive schedules (Figure 3g). The idle time increases with value of A for reactive schedules (Figure 3h).

Next, the impact of the size of ant population A on the solution robustness and quality robustness is investigated (Figure 3g,h). The following phenomenon can be noticed: The solution robustness of predictive schedules decreases with increasing value of ρ. After reaching the peak value for A = 15, the solution robustness increases with the value of A (Figure 3g). 

The size of ant population A has little impact on the quality robustness (Figure 3h).

**Figure 4:**
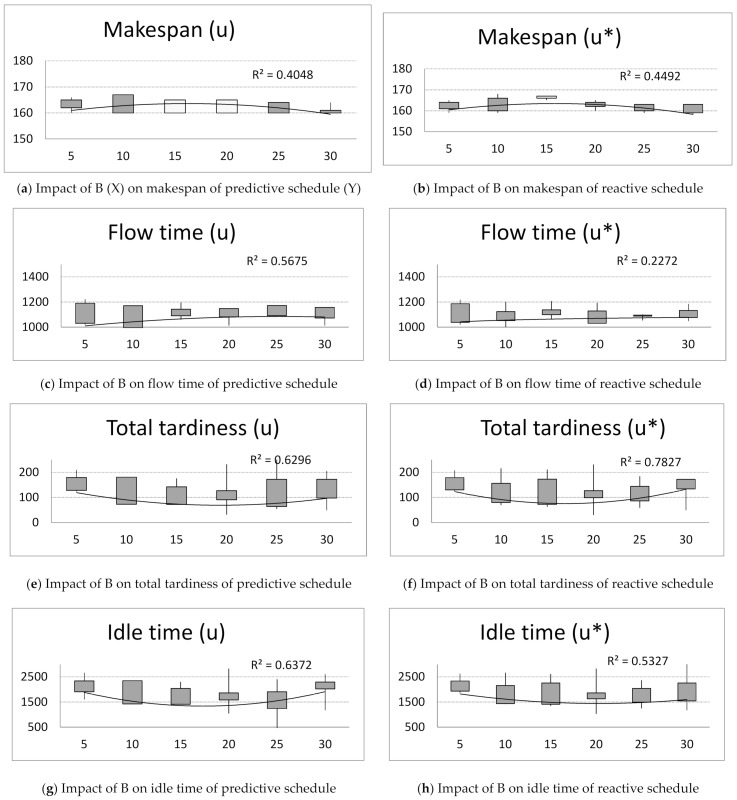

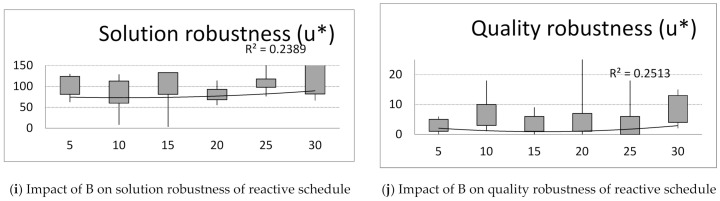
The impact of number of iterations, B = {5, 10, 15, 20, 25, 30} on makespan, flow time, total tardiness, and idle time of predictive schedules (u) and reactive schedules (u*), and solution robustness and quality robustness.

Next, computer simulations were run for unchanging number of ants A = 15, pheromone evaporation factor ρ = 0.7, parameter q = 0.5 and changing number of iterations, B = {5, 10, 15, 20, 25, 30}. Observing the achieved values of criteria: makespan, flow time, total tardiness and idle time for predictive and reactive schedules (Figure 4), the following conclusion can be drawn:

The makespan increases with increasing number of iterations, B. After reaching the peak value of B = 15, the makespan decreases with B for predictive (Figure 4a) and reactive schedules (Figure 4b).

The flow time of schedules increases with B for predictive (Figure 4b) and reactive schedules (Figure 4c).

The total tardiness decreases with B. After reaching the peak value of B = 20, the total delay increases with B (Figure 4e,f).

The idle time decreases with increasing value of B. After reaching the peak value of B = 20, the idle time increases with B for predictive (Figure 4g). and reactive schedules (Figure 4h).

Next, the impact of number of ants on the solution robustness and quality robustness is investigated. The solution robustness of predictive schedules slightly increases with increasing value of B. After reaching the peak value for B = 15, the solution robustness decreases with the value of B (Figure 4g). The same phenomenon is noticed for the quality robustness (Figure 4h).

**Figure 5:**
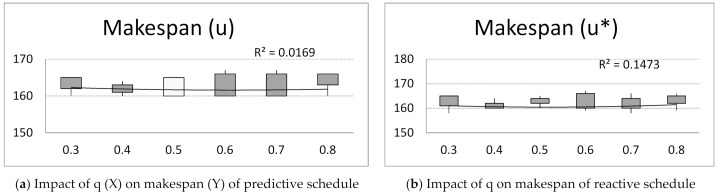

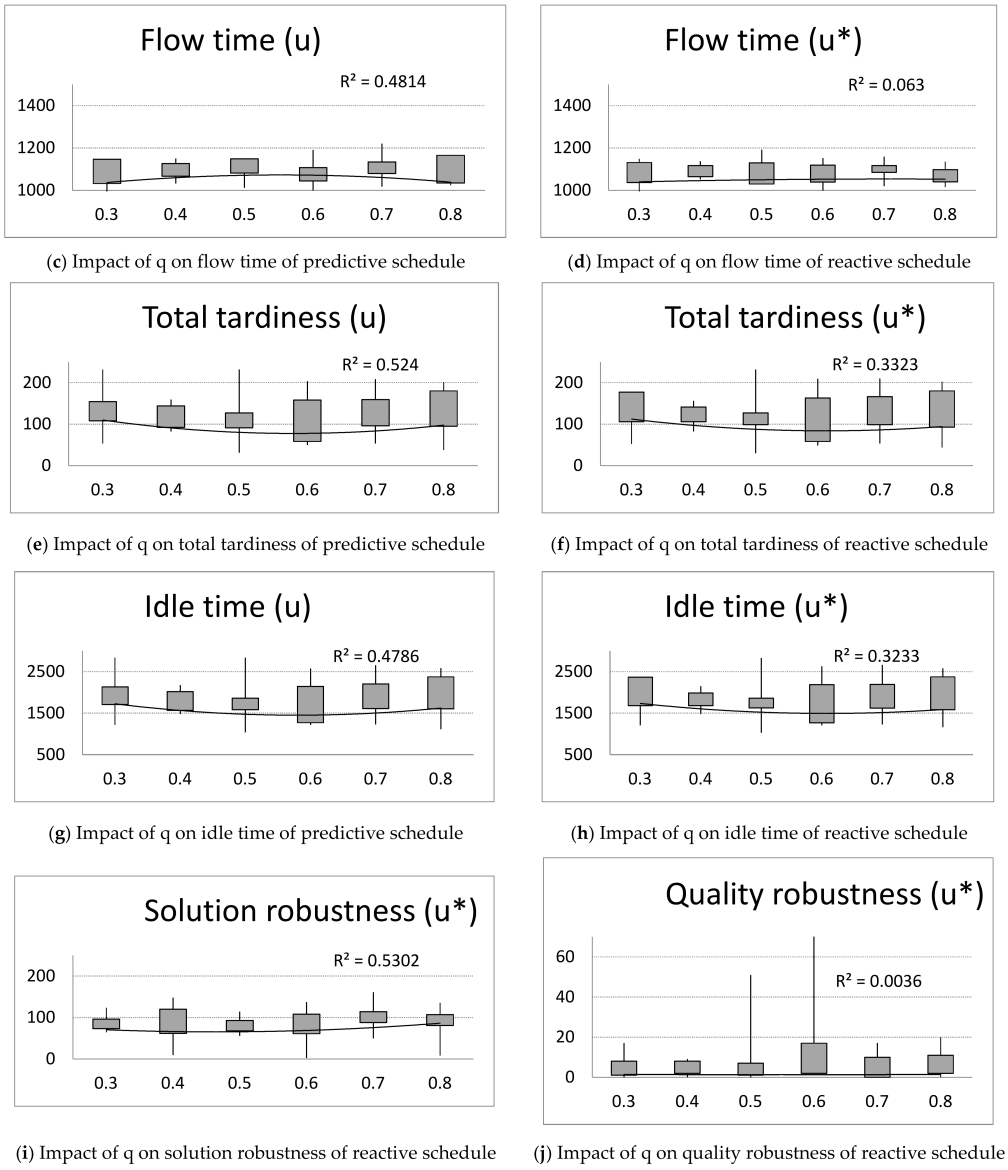
The impact of parameter q = {0.3, 0.4, 0.5, 0.6, 0.7, 0.8} on makespan, flow time, total tardiness, and idle time of predictive schedules (u) and reactive schedules (u*), and solution robustness and quality robustness.

Next, computer simulations were run for unchanging number of ants A= 15, number of iterations B = 20, pheromone evaporation factor ρ = 0.7 and changing parameter q = {0.3, 0.4, 0.5, 0.6, 0.7, 0.8}. Observing the achieved values of criteria: makespan, flow time, total tardiness and idle time for predictive and reactive schedules (Figure 5), the following conclusion can be drawn:

Parameter q_0_ has little impact on the makespan criterion of predictive schedules (Figure 5a). The makespan decreases with increasing parameter q. After reaching the peak value of q = 0.6, the makespan increases with q for reactive schedules (Figure 5b).

The flow time of schedules little increases with q. After reaching the peak value of q = 0.5, the flow time decreases with q for predictive schedules (Figure 5c). The flow time of schedules slightly increases with q for reactive schedules (Figure 5d).

The total tardiness decreases with increasing value of parameter q. After reaching the peak value of q = 0.5, the total delay increases with q (Figure 2f and Figure 5e).

The idle time decreases with increasing value of q. After reaching the peak value of q = 0.5, the idle time increases with q for schedules (Figure 2h and Figure 5g)

Additionally, the impact of parameter q on the solution robustness and quality robustness is investigated. The solution robustness of predictive schedules increases with increasing value of parameter q. After reaching the peak value for Parameter q = 0.5, the solution robustness decreases with the value of q (Figure 5g). Parameter q has little impact on the quality robustness (Figure 5h).

After the simulation, managers can gain knowledge about the key algorithm parameters that help achieve the best schedules for the following criteria: makespan, flow time, total tardiness and idle time for predictive and reactive schedules, solution robustness and quality robustness. Number of iterations has a strong influence over the above-mentioned criteria. The number of iterations is the most sensitive parameter. For example: the best values of makespan, total tardiness, idle times and solution robustness were achieved for parameter q = 0.5 (Figure 5).

Managers can gain knowledge about the impact of failure time of the FDM machine and the number of cases of noncompliance with the key process parameters of the standards on makespan, flow time, total tardiness and idle time of predictive and reactive schedules. For example, the total tardiness decreases with the increase in the disturbance-free operation of the FDM. After reaching the peak value of RDT = 58, the total delay increases with the failure-free time of the FDM machine (Figure 1). This information is important for predictive maintenance based not only on historical uptime information along with key-process-parameter conditions, but also proactive failure impact analysis.

## 7. Conclusions

The aim of the article was to develop a method for scheduling production and repair tasks along with an analysis of the historical failure times of FDM machine elements and deviations from the standards established for key process parameters: fill density, layer thickness and extruder temperature. Having physically observed the number of failures of the FDM machine, we defined weighted p-moving averages for the following periods and the equation for estimating the number of failures in the next planning period. Failure rate of the FDM machine was corrected with the probability of a stoppage in the future period due to the “outlier” in measurements of any of the key parameters of the additive process. Additionally, we investigated the impact of the failure time of the FDM machine and the number of cases of noncompliance with the key process parameters of the standards on criteria: makespan, total flow time, total delay, machine idle time, solution robustness and quality robustness. The presented model combined the practices of maintenance and production planning.

In future, firstly, we plan to analyze the model with a discrete time parameter and geometric probability distributions of failure-free times and repair times (see [26,27] for a survey of such models). In the next stage of the research, we plan to use the “working vacation” mechanism (proposed by Servi and Finn in [28]), in which the failed machine is not completely excluded from the task-servicing process, but the service process is slower and may be limited in scope. In the longest time horizon, it is assumed to consider a model in which the distribution of failure-free times and/or re-pair times will have a distribution without the “memoryless” property (i.e., other than exponential and geometric ones). Additionally, by analyzing the simulation results, it can be concluded that the effectiveness of the proposed ACO should be verified with other artificial-intelligence methods [29]. The proposed ant-colony optimization algorithm is only a solution search tool, thus the subject of future research will be the selection of an effective optimization method. Reliability characteristics and key process parameters of other machines, such as robots, will also be identified [30]. PdM periods will be estimated based on historical data and on-line information on key process parameters using various methods of statistical analysis.

## Figures and Tables

**Table 1:** The literature review on joint production and maintenance planning.

Type of Scheduling Problem	Policy of Maintenance	Optimization Algorithm	Objective Functions	Literature
two-machines flow shop	Constant- or variable-in-time machine failure rate and constant operating conditions	genetic algorithm	makespan	[14]
dynamic job shop	random machine failures	hybrid genetic and taboo search algorithm	makepan and schedule stability	[15]
permutation flow shop	predetermined periods of preventive maintenance with tolerance intervals around each maintenance period	Ant-colony optimization, genetic algorithm, tabu search and hybridization of these methods	delay, cost and quality functions and sustainability of production tools	[17]
maintenance department idle shops	insertion repair of emergent failure between the scheduled repair of regular failures in free margins	fuzzy goal programming	maximum number of repaired failures in the idle maintenance shops at any cost, maximum number of assigned emergent failure repairs	[18]
flexible flow shop manufacturing cells, sequence-dependent group scheduling problem	a machine level model describes machine reliability under group-varying conditions based on hazard rate	simulated annealing embedded genetic algorithm	preventive maintenance cost, minimal repair cost and job tardiness cost	[19]
flexible job shop	preventive maintenance based on well-chosen non-pre-emptive unavailability periods for preventive maintenance	dual-ants colony—a hybrid ant-colony optimization	makespan	[20]
	predicted probabilities of machine failures are assumed to be available	genetic algorithm	production gains and maintenance expenses (including profit per product, cost for maintenance and penalty for unscheduled maintenance)	[21]
permutation flow shops	insertion of the maintenance tasks is conducted according to several heuristics	Ant-colony optimization	makespan	[22]
parallel production system	the independent failure of single components, and the simultaneous common cause failure of all components		the sum of preventive and corrective maintenance costs, setup costs, holding costs, backorder costs and production costs	[23]
job shop with the FDM machine	maintenance periods estimated based on historical data on failure-free times of the FDM machine components and deviations from the standards established for the key process parameters	Ant-colony optimization	makespan, total flow time of production tasks, total delay of tasks, idle time of machines, schedule robustness and quality robustness	this paper

## Data Availability

All data contained within the article.
